# Correction: Protocol for the development of a procedure guide on Laparoscopic Cholecystectomy: Beyond bile duct injury prevention

**DOI:** 10.1371/journal.pone.0352181

**Published:** 2026-06-18

**Authors:** Camilo Ramírez-Giraldo, Daniela Álvarez-León, Alejandro Karduss-López, on behalf of Bogotá Task Force Collaboration Group

S2 Appendix is omitted from the list of Supporting Information. It can be viewed below.

## Supporting information

S2 Appendix Full list of contributors and institutional affiliations. (DOCX)
