# Supplementary material for: Protocol for the development of a procedure guide on Laparoscopic Cholecystectomy: Beyond bile duct injury prevention
Source: PLoS One. 2026 May 29;21(5):e0350562. doi: 10.1371/journal.pone.0350562 (PMC13220991; doi:10.1371/journal.pone.0350562)
Supplement: S1 Appendix — (DOCX) [file pone.0350562.s001.docx]

**S1 appendix.** Questions

1. **Key question:** In adult patients undergoing elective laparoscopic cholecystectomy, does the administration of perioperative prophylactic antibiotics reduce the incidence of surgical site infection and other postoperative infectious complications compared with no antibiotic administration?

| **Population (P)** | Adult patients undergoing elective laparoscopic cholecystectomy for benign gallbladder disease. |
| --- | --- |
| **Intervention (I)** | Administration of perioperative prophylactic antibiotics (within 120 minutes before surgical incision, single or multiple agents, any dosage regimen). |
| **Comparison (C)** | No antibiotic prophylaxis or placebo. |
| **Outcomes (O)** | 1. Incidence of surgical site infection.  2. Incidence of other postoperative infectious complications (e.g., pneumonia, urinary tract infection, sepsis).  3. Avoidance of unnecessary antibiotic use and prevention of bacterial resistance. 4. Adverse reactions related to antibiotic use. 5. Length of hospital stay and readmission rate. |

**Comment:** When we refer to perioperative antibiotic, it denotes the antibiotic administered within 120 minutes before the surgical incision, not the preoperative antibiotic, which refers to the one given in cases of acute cholecystitis while the surgical procedure is being performed.

1. **Key question:** In adult patients undergoing non-elective (urgent or emergency) laparoscopic cholecystectomy, reduce the incidence of surgical site infection and other postoperative infectious complications compared with no antibiotic administration?

| **Population (P)** | Adult patients undergoing non-elective laparoscopic cholecystectomy for benign gallbladder disease. |
| --- | --- |
| **Intervention (I)** | Administration of perioperative prophylactic antibiotics (within 120 minutes before surgical incision, single or multiple agents, any dosage regimen). |
| **Comparison (C)** | No antibiotic prophylaxis or placebo. |
| **Outcomes (O)** | 1. Incidence of surgical site infection. 2. Incidence of other postoperative infectious complications (e.g., pneumonia, urinary tract infection, sepsis). 3. Avoidance of unnecessary antibiotic use and prevention of bacterial resistance. 4. Adverse reactions related to antibiotic administration. 5. Length of hospital stay and readmission rate. |

1. **Key question:** In adult patients undergoing laparoscopic cholecystectomy, does intraoperative mechanical thromboprophylaxis reduce the risk of deep vein thrombosis and pulmonary embolism compared with no prophylaxis, without increasing adverse events?

| **Population (P)** | Adult patients undergoing laparoscopic cholecystectomy (elective or non-elective) for benign gallbladder disease. |
| --- | --- |
| **Intervention (I)** | Use of intraoperative mechanical thromboprophylaxis (e.g., graduated compression stockings, intermittent pneumatic compression devices, or venous foot pumps). |
| **Comparison (C)** | No mechanical prophylaxis or standard care without mechanical devices. |
| **Outcomes (O)** | 1. Incidence of deep vein thrombosis (DVT). 2. Incidence of pulmonary embolism (PE). 3. Procedure-related adverse events (e.g., skin lesions, nerve compression, hemodynamic effects). 4. Length of hospital stay and postoperative complications. |

1. **Key question:** In adult patients undergoing laparoscopic cholecystectomy, does subcutaneous infiltration of local anesthetic at trocar insertion sites, compared with no infiltration, reduce postoperative pain and analgesic requirements?

| **Population (P)** | Adult patients undergoing laparoscopic cholecystectomy (elective or non-elective) for benign gallbladder disease. |
| --- | --- |
| **Intervention (I)** | Subcutaneous infiltration of local anesthetic at trocar insertion sites, performed before or after trocar placement. |
| **Comparison (C)** | No local anesthetic infiltration or placebo (saline infiltration). |
| **Outcomes (O)** | 1. Postoperative pain intensity (measured by visual analog scale or numeric rating scale). 2. Postoperative analgesic consumption. 3. Time to first analgesic request. 4. Length of hospital stay and recovery time. 5. Adverse events related to anesthetic use (e.g., local toxicity, allergic reaction). |

**Comment:** For the purpose of this guideline, local anesthetic infiltration refers to the subcutaneous administration of an anesthetic agent (e.g., lidocaine, bupivacaine, or ropivacaine) at the trocar insertion sites, performed either before or after trocar placement.
This intervention aims to reduce somatic pain derived from trocar incisions without affecting visceral pain. The technique excludes the use of intraperitoneal, port-site block, or regional anesthesia.

1. **Key question:** In adult patients undergoing laparoscopic cholecystectomy, does the open technique for initial trocar insertion, compared with the Veress needle or direct optical entry, reduce access-related complications without increasing operative time or postoperative morbidity?

| **Population (P)** | Adult patients undergoing laparoscopic cholecystectomy (elective or non-elective) for benign gallbladder disease. |
| --- | --- |
| **Intervention (I)** | Initial insertion of the first trocar using the open technique (e.g., Hasson or open cut-down approach). |
| **Comparison (C)** | Closed Veress needle technique or direct optical trocar entry for pneumoperitoneum creation and initial trocar placement. |
| **Outcomes (O)** | 1. Incidence of access-related complications (e.g., vascular or bowel injury, gas embolism, subcutaneous emphysema). 2. Time required to establish pneumoperitoneum. 3. Conversion to open surgery. 4. Postoperative port-site pain or infection. 5. Mortality and major morbidity. |

**Comment:** For the purpose of this guideline:

- The open technique refers to the Hasson or open cut-down approach, in which the peritoneal cavity is entered under direct vision before placement of the first trocar.
- The closed technique (Veress needle) involves blind peritoneal access using a spring-loaded Veress needle to create pneumoperitoneum prior to trocar insertion.
- The direct optical entry technique uses a trocar with an integrated optical system, allowing continuous visualization of tissue layers during insertion without prior insufflation.

These three techniques represent the main approaches for establishing pneumoperitoneum in laparoscopic surgery. The choice of method should be based on the surgeon’s experience, patient characteristics, and history of previous abdominal surgery.

1. **Key question:** In adult patients with prior abdominal surgery undergoing laparoscopic cholecystectomy, does placing the first trocar away from previous scars, compared with standard umbilical insertion, reduce access-related complications without prolonging surgery or increasing conversion rates?

| **Population (P)** | Adult patients with a history of previous abdominal surgery undergoing laparoscopic cholecystectomy (elective or non-elective) for benign gallbladder disease. |
| --- | --- |
| **Intervention (I)** | Initial insertion of the first trocar at a site distant from the previous surgical scar (e.g., Palmer’s point, left upper quadrant, or alternative non-umbilical entry). |
| **Comparison (C)** | Standard umbilical trocar insertion for pneumoperitoneum creation and access. |
| **Outcomes (O)** | 1. Incidence of access-related complications (e.g., vascular or bowel injury, gas embolism, subcutaneous emphysema). 2. Operative time. 3. Conversion to open surgery. 4. Port-site infection or postoperative pain. 5. Mortality or major morbidity. |

**Comment:** For the purpose of this guideline, non-umbilical entry refers to alternative access sites used in patients with previous abdominal surgery, where periumbilical adhesions are suspected.
Common approaches include **Palmer’s point** (3 cm below the left costal margin in the midclavicular line), **Jain point** (2–3 cm below and lateral to Palmer’s point) or **Lee–Huang point** (located on the midline, midway between the xiphoid process and the umbilicus).

The choice of entry site should depend on the patient’s surgical history, body habitus, and the surgeon’s experience, ensuring safe visualization and minimizing the risk of bowel or vascular injury.

1. **Key question:** In adult patients undergoing laparoscopic cholecystectomy, does the use of fewer ports (one, two, or three) compared with the standard four-port technique reduce operative time and postoperative pain without increasing conversion rates or surgical complications?

| **Population (P)** | Adult patients undergoing laparoscopic cholecystectomy (elective or non-elective) for benign gallbladder disease. |
| --- | --- |
| **Intervention (I)** | Use of a reduced-port technique — one-, two-, or three-port laparoscopic cholecystectomy. |
| **Comparison (C)** | Standard four-port laparoscopic cholecystectomy. |
| **Outcomes (O)** | 1. Operative time. 2. Postoperative pain intensity (measured by VAS or NRS). 3. Analgesic consumption. 4. Bail-out procedures. 5. Surgical complications (e.g., bile duct injury, bleeding, infection). 6. Length of hospital stay.  7. Cosmetic or aesthetic outcome (patient satisfaction with scar appearance, body image, or cosmetic score). |

1. **Key question:** In adult patients undergoing laparoscopic cholecystectomy, does the American position, compared with the French position, reduce operative time, improve surgical ergonomics, and maintain procedural safety?

| **Population (P)** | Adult patients undergoing laparoscopic cholecystectomy (elective or non-elective) for benign gallbladder disease. |
| --- | --- |
| **Intervention (I)** | American position — patient in supine position with both legs together; the surgeon stands on the patient’s left side, the assistant on the right, and the camera operator to the surgeon’s left. |
| **Comparison (C)** | French position — patient in lithotomy position with legs apart; the surgeon stands between the patient’s legs, with assistants on each side. |
| **Outcomes (O)** | 1. Operative time. 2. Surgical ergonomics (comfort, posture, hand strain, and fatigue). 3. Intraoperative complications (e.g., bile duct injury, bleeding, trocar injury). 4. Bail-out procedures. 5. Postoperative complications and hospital stay. 6. Overall procedural safety and surgeon performance. |

**Comment:** For the purpose of this guideline:

- The American position refers to the setup in which the patient is placed in a supine position with both legs together, the surgeon stands on the patient’s left side, the assistant on the right, and the camera operator to the surgeon’s left. This configuration is commonly used in North and South America and is ergonomically favorable for right-handed surgeons.
- The French position involves the patient in a lithotomy position with legs apart, with the surgeon standing between the patient’s legs and assistants on both sides. It allows central access to the operative field and can facilitate bimanual coordination, particularly in teaching environments.

1. **Key question:** In adult patients undergoing laparoscopic cholecystectomy, does the use of the supine position with reverse Trendelenburg and left lateral tilt, compared with the flat supine position, improve surgical exposure and reduce operative time without increasing intraoperative complications or physiological adverse effects?

| **Population (P)** | Adult patients undergoing laparoscopic cholecystectomy (elective or non-elective) for benign gallbladder disease. |
| --- | --- |
| **Intervention (I)** | Supine position with reverse Trendelenburg (head-up tilt) and left lateral rotation (tilt) to improve visualization of the gallbladder and Calot’s triangle. |
| **Comparison (C)** | Flat supine position without reverse Trendelenburg or left tilt. |
| **Outcomes (O)** | 1. Quality of surgical exposure (subjective or objective assessment). 2. Operative time. 3. Intraoperative complications (e.g., bile duct injury, bleeding, trocar injury). 4. Physiological effects (e.g., hemodynamic changes, CO₂ retention, respiratory mechanics). 5. Bail-out procedures. 6. Postoperative recovery and complications. |

1. **Key question:** In adult patients undergoing laparoscopic cholecystectomy (elective or non-elective) for benign gallbladder disease, does the use of 5-mm trocars for all ports combined with a 5-mm laparoscope, compared with the standard technique employing a 12-mm umbilical and/or epigastric trocar, reduce postoperative pain and access-related complications without compromising surgical safety or increasing operative time?

| **Population (P)** | Adult patients undergoing laparoscopic cholecystectomy (elective or non-elective) for benign gallbladder disease. |
| --- | --- |
| **Intervention (I)** | Use of exclusively 5-mm trocars for all ports with a 5-mm laparoscope. |
| **Comparison (C)** | Standard technique using a 12-mm umbilical and/or epigastric trocar. |
| **Outcomes (O)** | 1. Quality of surgical exposure (subjective or objective assessment). 2. Operative time. 3. Intraoperative complications (e.g., bile duct injury, bleeding, trocar injury). 4. Reduction in postoperative pain. 5. Bail-out procedures. 6. Postoperative recovery and complications.  7. Reduction in access-site complications |

1. **Key question:** In adult patients undergoing laparoscopic cholecystectomy (elective or non-elective) for benign gallbladder disease, does the use of a 0°, 30°, or 45° laparoscope, compared with alternative laparoscopic viewing angles, improve intraoperative safety and visualization without increasing operative time or complication rates?

| **Population (P)** | Adult patients undergoing laparoscopic cholecystectomy (elective or non-elective) for benign gallbladder disease. |
| --- | --- |
| **Intervention (I)** | Use of a specific laparoscope angle (0°, 30°, or 45°) |
| **Comparison (C)** | Use of alternative laparoscopic viewing angles (e.g., 0° vs 30° vs 45°) |
| **Outcomes (O)** | 1. Quality of surgical exposure (subjective or objective assessment). 2. Operative time. 3. Intraoperative complications (e.g., bile duct injury, bleeding, trocar injury). 4. Bail-out procedures. 5. Postoperative recovery and complications. |

1. **Key question:** In adult patients undergoing laparoscopic cholecystectomy, does transabdominal fundus retraction using a suture, compared with the standard four-port technique, reduce operative time and postoperative pain while improving cosmetic outcomes and minimizing access-site complications and conversion rates, without compromising overall surgical safety?

| **Population (P)** | Adult patients undergoing laparoscopic cholecystectomy (elective or non-elective) for benign gallbladder disease. |
| --- | --- |
| **Intervention (I)** | Transabdominal fundus retraction using a suture. |
| **Comparison (C)** | Standard four-port laparoscopic cholecystectomy technique. |
| **Outcomes (O)** | 1.Reduced operative time  2. Improved postoperative pain  3. Maintenance of surgical safety  4. Cosmetic outcomes / patient satisfaction  5. Access-site complications (e.g., bleeding, infection, port-site hernia)  6. Bail-out procedures. |

1. **Key question:** In adult patients with cardiovascular comorbidity undergoing laparoscopic cholecystectomy, does the use of low-pressure pneumoperitoneum (e.g., 8–10 mmHg), compared with standard-pressure pneumoperitoneum (12–15 mmHg), reduce postoperative complications while maintaining adequate intraoperative visualization and surgical performance? Additionally, does low-pressure pneumoperitoneum decrease intraoperative hemodynamic events or the need for vasoactive support, without increasing operative time or conversion rates due to reduced exposure?

| **Population (P)** | Adult patients with cardiovascular comorbidity undergoing laparoscopic cholecystectomy (elective or non-elective) for benign gallbladder disease. |
| --- | --- |
| **Intervention (I)** | Low-pressure CO₂ pneumoperitoneum (approximately 8–10 mmHg). |
| **Comparison (C)** | Standard-pressure CO₂ pneumoperitoneum (12–15 mmHg). |
| **Outcomes (O)** | 1.Reduction in postoperative complications  2. Adequate intraoperative visualization (non-inferior to standard pressure)  3. Reduction in intraoperative hemodynamic instability  4. Reduced need for vasoactive support  5. No increase in operative time  6. Bail-out procedures due to inadequate exposure |

1. **Key question:** In adult patients undergoing laparoscopic cholecystectomy, which intraoperative difficulty score—such as the Nassar scale, Parkland grading scale, AAST cholecystitis severity score, or other validated grading systems—provides the most reliable, reproducible, and clinically useful assessment of operative difficulty to support standardized reporting, comparative research, and surgical audit?

| **Population (P)** | Adult patients undergoing laparoscopic cholecystectomy (elective or non-elective) for benign gallbladder disease. |
| --- | --- |
| **Index test (I)** | Use of a specific intraoperative difficulty grading system (e.g., Nassar, Parkland, AAST, or other validated scales. |
| **Reference standard (R)** | 1.Operative difficulty (as judged by expert surgeons)  2. Need for bail-out procedures  3. Operative time  4. Intraoperative complications |
| **Target condition(D)** | Accurate classification of intraoperative difficulty during laparoscopic cholecystectomy |

**Comment:** It is important to clearly differentiate intraoperative difficulty grading systems from preoperative prediction scores. Preoperative scales aim to estimate the likelihood of a difficult cholecystectomy based on clinical, laboratory, or imaging findings, and are used primarily for planning and risk stratification. In contrast, intraoperative scoring systems objectively assess the actual technical difficulty encountered during surgery. These intraoperative tools provide a standardized framework for documenting operative complexity, supporting comparative studies, surgical audit, training assessment, and guideline development. Therefore, distinguishing between predictive (preoperative) and descriptive (intraoperative) scales is essential to ensure appropriate application and methodological clarity.

1. **Key question:** In adult patients undergoing laparoscopic cholecystectomy, does controlled gallbladder drainage or decompression (via needle puncture and aspiration) before initiating dissection, compared with no prior gallbladder decompression, reduce the bail-out procedures, bile duct injury, or surgical site infection (intraabdoinal abscess), while also influencing operative time and length of hospital stay?

| **Population (P)** | Adult patients undergoing laparoscopic cholecystectomy (elective or non-elective) for benign gallbladder disease. |
| --- | --- |
| **Intervention (I)** | Controlled gallbladder drainage/decompression (needle puncture and aspiration) prior to beginning dissection. |
| **Comparison (C)** | No gallbladder drainage/decompression prior to dissection. |
| **Outcomes (O)** | 1. Bail-out procedures  2. Bile duct injury  3. Surgical site infection  4. Operative time  5. Length of hospital stay |

**16. Key question:** In adult patients undergoing laparoscopic cholecystectomy, does obtaining intraoperative bile cultures, compared with not obtaining bile cultures, improve postoperative infectious outcomes or guide more appropriate antibiotic therapy without increasing costs or unnecessary antibiotic use?

| **Population (P)** | Adult patients undergoing laparoscopic cholecystectomy (elective or non-elective) for benign gallbladder disease. |
| --- | --- |
| **Intervention (I)** | Routine or selective intraoperative bile culture. |
| **Comparison (C)** | No intraoperative bile culture. |
| **Outcomes (O)** | 1. Surgical site infection  2. Need for change or escalation of antibiotics  3. Costs related to microbiology and antibiotic use  4. Antibiotic overuse / antimicrobial resistance risk  5. Length of hospital stay |

**17. Key question:** In adult patients undergoing laparoscopic cholecystectomy, does infundibular dissection for identification and control of the cystic duct, compared with the fundus-first technique (top-down technique), reduce the incidence of bile duct injury and influence other operative and postoperative outcomes?

| **Population (P)** | Adult patients undergoing laparoscopic cholecystectomy (elective or non-elective) for benign gallbladder disease. |
| --- | --- |
| **Intervention (I)** | Infundibular dissection for identification and control of the cystic duct. |
| **Comparison (C)** | Fundus-first technique (top-down technique) |
| **Outcomes (O)** | 1. Intraoperative complications (e.g., bile duct injury, bleeding, trocar injury). 2. Bail-out procedures. 3. Postoperative recovery and complications.  4. Operative time. |

**18. Key question:** In adult patients undergoing laparoscopic cholecystectomy, does dissection of the hepatocystic triangle using monopolar energy, compared with blunt and/or sharp dissection without monopolar energy as the primary method, affect the incidence of bile duct injury, biliary stricture, or operative time?

| **Population (P)** | Adult patients undergoing laparoscopic cholecystectomy (elective or non-elective) for benign gallbladder disease. |
| --- | --- |
| **Intervention (I)** | Dissection of the hepatocystic triangle using monopolar energy. |
| **Comparison (C)** | Blunt and/or sharp dissection (e.g., scissors) without monopolar energy as the main technique. |
| **Outcomes (O)** | 1. Intraoperative complications (e.g., bile duct injury, bleeding). 2. Biliary stricture.  3. Operative time. |

**19. Key question:** In adult patients undergoing laparoscopic cholecystectomy, does the use of intraoperative irrigation—whether for lavage or hydrodissection—compared with no irrigation, improve the identification of critical structures and reduce the risk of bile duct injury, while affecting postoperative intra-abdominal collections, operative time, bleeding, conversion rates, and other procedure-related complications?

| **Population (P)** | Adult patients undergoing laparoscopic cholecystectomy (elective or non-elective) for benign gallbladder disease. |
| --- | --- |
| **Intervention (I)** | Use of intraoperative irrigation (for lavage or hydrodissection) during dissection of the hepatocystic triangle or gallbladder bed. |
| **Comparison (C)** | No irrigation during the procedure. |
| **Outcomes (O)** | 1. Postoperative intra-abdominal collections.  2. Intraoperative complications (e.g., bile duct injury, bleeding). 3. Bail-out procedures.  4. Operative time.  5. Quality of identification of critical structures. |

**20. key question:** In adult patients undergoing laparoscopic cholecystectomy, does the use of routine intraoperative biliary imaging—such as indocyanine green fluorescence imaging, intraoperative ultrasound, or routine intraoperative cholangiography—compared with standard white-light visualization alone, reduce the incidence of bile duct injury, bail-out procedures, or undiagnosed choledocholithiasis, while minimizing operative time, postoperative interventions, and overall costs?

| **Population (P)** | Adult patients undergoing laparoscopic cholecystectomy (elective or non-elective) for benign gallbladder disease. |
| --- | --- |
| **Intervention (I)** | Routine use of advanced intraoperative biliary imaging, including:   - Indocyanine green fluorescence cholangiography - Intraoperative ultrasound - Routine intraoperative cholangiography |
| **Comparison (C)** | Standard white-light visualization without routine intraoperative imaging. |
| **Outcomes (O)** | 1. Intraoperative complications (e.g., bile duct injury). 2. Bail-out procedures.  3. Rate of successful identification of biliary anatomy.  4. Operative time.  5. Surgeon confidence/visualization quality (validated scales). |

**21. Key question:** In adult patients undergoing laparoscopic cholecystectomy, does the systematic use and documentation of the Critical View of Safety, compared with alternative methods of anatomic identification or non-standardized dissection techniques, reduce the incidence of bile duct injury and the need for bailout procedures, while affecting operative time, postoperative complications, and surgeon-reported confidence in biliary anatomy identification?

| **Population (P)** | Adult patients undergoing laparoscopic cholecystectomy (elective or non-elective) for benign gallbladder disease. |
| --- | --- |
| **Intervention (I)** | Systematic use and documentation of the Critical View of Safety for identification of hepatocystic anatomy prior to clipping and division of the cystic duct and artery. |
| **Comparison (C)** | Alternative or non-standardized techniques of anatomic identification, such as:   - Infundibular (duct–artery) technique - Fundus-first (top-down) approach - Anatomical identification without fulfillment of CVS criteria - Surgeon’s subjective identification without a formal checklist |
| **Outcomes (O)** | 1. Bile duct injury. 2. Intraoperative complications.  3. Postoperative complications.  4. Operative time.  5. Surgeon-reported confidence in biliary anatomy identification. |

**22. key question:** In adult patients undergoing laparoscopic cholecystectomy in whom an adequate Critical View of Safety cannot be achieved, which bail-out strategy—such as laparoscopic subtotal cholecystectomy (fenestrating or reconstituting), fundus-first dissection, conversion to open surgery, intraoperative cholecystostomy, or abandon procedure—provides the safest anatomical control and lowers the risk of bile duct injury compared? Additionally, how do these bail-out approaches affect postoperative morbidity, need for reintervention, and overall recovery?

| **Population (P)** | Adult patients undergoing laparoscopic cholecystectomy (elective or non-elective) for benign gallbladder disease in whom the Critical View of Safety cannot be safely achieved. |
| --- | --- |
| **Intervention (I)** | Systematic use and documentation of the Critical View of Safety for identification of hepatocystic anatomy prior to clipping and division of the cystic duct and artery. |
| **Comparison (C)** | Bail-out procedures, including:   - Laparoscopic subtotal cholecystectomy (fenestrating or reconstituting). - Fundus-first (top-down) dissection. - Conversion to open surgery. - Intraoperative cholecystostomy. - Abandon procedure |
| **Outcomes (O)** | 1. Incidence of bile duct injury 2. Overall postoperative morbidity and mortality.  3. Need for reintervention.  4. Length of hospital stay.  5. Operative time.  6. Long-term functional outcomes (biliary strictures, recurrent symptoms) |

**Comment:** When specifying bail-out procedures in laparoscopic cholecystectomy, it is important to clarify the role of conversion to open surgery. Preemptive conversion—also referred to as elective or strategic conversion—is a deliberate decision to convert before any intraoperative complication occurs, usually because the anatomy cannot be safely defined or the Critical View of Safety cannot be achieved. In this context, preemptive conversion is considered a valid bail-out procedure, as its purpose is to prevent injury by transitioning to a safer operative environment.

In contrast, reactive conversion, performed only after a complication has already developed (such as bleeding, bile duct injury, or loss of control of the operative field), is not considered a bail-out strategy, but rather an emergency response to an adverse event. Reactive conversion does not prevent complications—it manages their consequences.

Therefore, in the context of this question, “conversion to open ” refers exclusively to preemptive conversion as a bail-out option, not reactive conversion.

**23. key question:** In adult patients undergoing laparoscopic cholecystectomy in whom a subtotal cholecystectomy is required as a bail-out procedure, does the reconstituting technique, compared with the fenestrating technique, reduce postoperative bile leak, complications, and the need for reintervention, while affecting long-term biliary outcomes, operative time, and risk of recurrent biliary symptoms?

| **Population (P)** | Adults undergoing laparoscopic cholecystectomy who require a subtotal cholecystectomy as a bail-out technique due to inability to obtain a safe Critical View of Safety. |
| --- | --- |
| **Intervention (I)** | Subtotal cholecystectomy – reconstituting technique (closing the remnant gallbladder stump and restoring a pouch). |
| **Comparison (C)** | Subtotal cholecystectomy – fenestrating technique (leaving the remnant open, with or without mucosectomy or drainage). |
| **Outcomes (O)** | 1. Overall postoperative morbidity and mortality.  2. Need for reintervention (ERCP or drainage).  3. Postoperative bile leak.  4. Length of hospital stay.  5. Operative time.  6. Recurrent biliary symptoms or need for completion cholecystectomy) |

**24. Key question:** In adult patients undergoing laparoscopic cholecystectomy, does the use of advanced energy devices (such as ultrasonic shears, bipolar vessel-sealing systems, or other advanced energy platforms) for cystic duct and cystic artery control, compared with standard metallic clips, improve the safety of ductal and arterial closure—by reducing bile leakage and arterial stump bleeding—while also affecting operative time, conversion rates, postoperative complications or cost?

| **Population (P)** | Adult patients undergoing laparoscopic cholecystectomy (elective or non-elective) for benign gallbladder disease. |
| --- | --- |
| **Intervention (I)** | Use of advanced energy devices for cystic duct and cystic artery control, including:   - Ultrasonic energy devices - Bipolar vessel-sealing systems - Advanced energy platforms capable of sealing ducts and vessels |
| **Comparison (C)** | Standard metallic clips for cystic duct and cystic artery control. |
| **Outcomes (O)** | 1. Bile leakage. 2. Coversion to open.  3. Cost of the procedure.  4. Operative time. |

**25. Key question:** In adult patients undergoing laparoscopic cholecystectomy who develop intraoperative bleeding, which hemostatic strategy—such as advanced energy devices, clips or endoloops, laparoscopic suturing, or topical hemostatic agents with compression—provides the safest and most effective control of hemorrhage, compared with alternative methods, in terms of achieving hemostasis, preventing rebleeding, reducing conversion to open surgery, and minimizing method-related complications? Furthermore, does the preferred hemostatic approach differ between minor bleeding from the gallbladder bed and significant vascular bleeding?

| **Population (P)** | Adults undergoing laparoscopic cholecystectomy who experience intraoperative bleeding, including:   - Minor bleeding from the gallbladder bed or dissection planes - Major bleeding from identifiable vessels (e.g., cystic artery, hepatic arterial branches, venous plexus) |
| --- | --- |
| **Intervention (I)** | Use of **specific hemostatic techniques,** including**:**   - Advanced energy devices (ultrasonic shears, bipolar vessel sealers) - Clips or endoloops - Laparoscopic suturing - Topical hemostatic agents and compression (e.g., Surgicel, Floseal, gauze + electrocautery) |
| **Comparison (C)** | Any alternative hemostatic method, such as:   - Clips vs. energy devices - Energy devices vs. suturing - Suturing vs. topical hemostasis - Compression/electrocautery alone vs. mechanical control (Comparisons may differ depending on whether bleeding is minor or major.) |
| **Outcomes (O)** | 1. Success of intraoperative hemostasis. 2. Rebleeding requiring further intervention.  3. **Conversion to open surgery** due to uncontrolled bleeding.  4. Operative time.  5. Need for blood transfusion.  6. Postoperative complications.  7. Length of hospital stay. |

**25. Key question:** In adult patients undergoing laparoscopic cholecystectomy, does routine placement of an abdominal drain at the end of the procedure, compared with no routine drainage, reduce postoperative bile-related or hemorrhagic complications—such as biloma, bile leak, or intra-abdominal abscess—and the need for reintervention, while avoiding increases in postoperative pain, length of hospital stay, drain-related morbidity, and impaired recovery?

| **Population (P)** | Adult patients undergoing laparoscopic cholecystectomy (elective or non-elective) for benign gallbladder disease. |
| --- | --- |
| **Intervention (I)** | Routine placement of an abdominal drain at the end of the procedure. |
| **Comparison (C)** | No routine placement of an abdominal drain. |
| **Outcomes (O)** | 1. Bile leakage. 2. Biloma or intra-abdominal abscess formation.  3. Need for reintervention.  4. Postoperative pain.  5. Length of hospital stay  6. **Drain-related complications** (infection at insertion site, accidental dislodgement, irritation) |

**26. Key question:** In adult patients undergoing laparoscopic cholecystectomy, does extracting the gallbladder through the umbilical port, compared with extraction through the epigastric port, reduce the risk of port-site infection and port-site hernia, while affecting postoperative pain, intraoperative gallbladder rupture or bile spillage, operative time (including need for incision enlargement or change of extraction site), cosmetic outcomes, and abdominal wall complications?

| **Population (P)** | Adult patients undergoing laparoscopic cholecystectomy (elective or non-elective) for benign gallbladder disease. |
| --- | --- |
| **Intervention (I)** | Extraction of the gallbladder through the umbilical port. |
| **Comparison (C)** | Extraction of the gallbladder through the epigastric port. |
| **Outcomes (O)** | 1. Port-site surgical site infection. 2. Port-site hernia.  3. Bile spillage or gallbladder rupture during extraction.  4. Localized postoperative pain at extraction site.  5. Need for incision enlargement.  6. Wound complications: hematoma, seroma and dehiscence. |

**27. Key question:** In adult patients undergoing laparoscopic cholecystectomy, does the use of any protective extraction device—whether a commercially manufactured retrieval bag or an improvised device—compared with extracting the gallbladder without any protective device, reduce the risk of surgical site infection, bile or stone spillage, and gallbladder rupture, while influencing operative ease, extraction time, and oncologic safety in cases of incidental neoplasia?

| **Population (P)** | Adult patients undergoing laparoscopic cholecystectomy (elective or non-elective) for benign gallbladder disease. |
| --- | --- |
| **Intervention (I)** | Use of any protective extraction device, including:   - Commercial laparoscopic retrieval bags, or - Improvised extraction devices (glove-finger bags, sterile IV-fluid bags, etc.). |
| **Comparison (C)** | No extraction device used during gallbladder retrieval. |
| **Outcomes (O)** | 1. Surgical site infection. 2. Bile spillage during extraction  3. Gallstone dissemination in the peritoneal cavity.  4. Intraoperative gallbladder rupture.  5. Safety in incidental gallbladder cancer (risk of peritoneal seeding).  6. Need for incision enlargement.  7. Extraction time.  8. Cost. |

**28. Key question:** In adult patients undergoing laparoscopic cholecystectomy, does the instillation or irrigation of local anesthetic (such as bupivacaine, ropivacaine, or lidocaine) into the gallbladder bed at the end of the procedure, compared with no local anesthetic infiltration, reduce immediate postoperative pain and improve early recovery while avoiding local or systemic adverse effects?

| **Population (P)** | Adult patients undergoing laparoscopic cholecystectomy (elective or non-elective) for benign gallbladder disease. |
| --- | --- |
| **Intervention (I)** | Instillation or irrigation of a local anesthetic into the gallbladder bed at the end of surgery (e.g., bupivacaine, ropivacaine, lidocaine). |
| **Comparison (C)** | No instillation of local anesthetic in the gallbladder bed (standard analgesic management alone). |
| **Outcomes (O)** | 1. Intensity of immediate postoperative pain, measured by validated scales. 2. P**ostoperative analgesic consumption** (opioids and non-opioids).  3. Length of hospital stay.  4. Local anesthetic adverse events.  5. Patient satisfaction with pain control.  6. Need for unplanned medical evaluation due to pain. |
