# Supplementary material for: Protocol for the development of a procedure guide on Laparoscopic Cholecystectomy: Beyond bile duct injury prevention
Source: PLoS One. 2026 May 29;21(5):e0350562. doi: 10.1371/journal.pone.0350562 (PMC13220991; doi:10.1371/journal.pone.0350562)
Supplement: Attachment — Submitted filename: Response to Reviewers.docx [file pone.0350562.s002.docx]

**Point-by-point response to reviewers´**

Thank you for the valuable feedback and constructive reviewers’ comments aimed at improving this study.

**Reviewer 1**

1. Comment: Overall, I believe the manuscript is suitable for acceptance in its current form. The only minor revision I would suggest is to reconsider the wording in the sentence “despite its routine nature, laparoscopic cholecystectomy is not without risk” (line 105). The term “routine” may inadvertently convey a sense of triviality, which contrasts with the manuscript’s focus on patient safety. A more neutral alternative could be: “Despite being frequently performed, laparoscopic cholecystectomy is not without risk.”

Answer: We sincerely thank the reviewer for the kind and thoughtful comments, as well as for the positive overall assessment of our manuscript. We agree with this suggestion and have revised the sentence accordingly. The text now reads: “Despite being frequently performed, laparoscopic cholecystectomy is not without risk.”

**Reviewer 2**

1. Comment: Use of ChatGPT in literature search strategy (key issue): On page 12, the authors mention, "Systematic literature searches will be performed with the assistance of ChatGPT 5.0." This is a serious methodological flaw and does not comply with the standards of systematic reviews. Using generative AI tools for systematic literature searches is unreliable and may lead to hallucinations (producing false literature), omissions, and reproducibility issues. PLOS ONE requires methods to be transparent and reproducible. This method must be removed and replaced with standard, reportable search strategies. Suggestion: Provide a detailed description of the search databases (such as PubMed/MEDLINE, Embase, Cochrane Library), search terms (including MeSH terms and free terms), search time frame, language restrictions, etc. It can be mentioned that professional medical librarians will be consulted to ensure the comprehensiveness of the search.

Answer: We thank the reviewer for this important comment. We agree that the original wording may have suggested that generative AI would be used to conduct the systematic searches themselves, which was not our intention. We have therefore revised the manuscript to clarify that all final literature searches will be conducted using conventional, reproducible search strategies in PubMed/MEDLINE, Embase, and the Cochrane Library. For each clinical question, generative AI may be used only to assist in drafting preliminary search strings, which will then be critically reviewed, refined, and validated by the investigators before implementation. We have revised the Methods section accordingly to make the search process more transparent and reproducible.

2. Comment: Consensus threshold: Setting "≥50% participation rate and ≥80% agreement rate" as the consensus standard is reasonable. However, it needs to be clarified how to handle cases where the participation rate is exactly on the 50% edge (for example, whether reminders will be sent after the first round to increase participation).

Answer: We thank the reviewer for this helpful suggestion. We agree that the procedure for handling participation at the threshold should be clarified. We have revised the manuscript to specify that reminder notifications will be sent to participants during each Delphi round to optimize response rates. We also clarify that a participation rate of exactly 50% will be considered acceptable for that round, consistent with the predefined threshold of ≥50%. If participation falls below 50%, the round will remain open for an additional reminder period before finalizing the results. This clarification has been added to the Methods section.

3. Comment: Composition and representativeness of the expert panel: Although a large number of experts (about 60) are listed, the criteria for expert selection (such as based on publication records, years of clinical experience, regional representation, etc.) need to be described in more detail to demonstrate their professionalism. Additionally, consideration should be given to including methodologists, patient representatives, or nursing representatives to enhance the comprehensiveness and applicability of the guidelines (AGREE II domain 3).

Answer: We thank the reviewer for this thoughtful and important comment. We agree that the criteria for expert selection should be described in greater detail to better demonstrate the expertise and representativeness of the panel. We have therefore revised the manuscript to clarify that invited participants will be selected based on a combination of factors, including recognized clinical expertise in laparoscopic cholecystectomy or hepatobiliary surgery, academic and/or research experience, years of professional practice, and the aim of ensuring representation across different institutions, healthcare settings, surgical subspecialties, and geographic regions.

We also agree on the importance of multidisciplinary input. In response, we have made the role of methodological support more explicit in the manuscript and clarified that anesthesiologists were invited for questions specifically related to perioperative analgesia and anesthetic management. Because the present guideline is intentionally focused on intraoperative technical conduct, patient and nursing participation was not included in the initial Delphi panel; however, we acknowledge that these perspectives may be valuable for future implementation, dissemination, and updating phases of the guideline. We appreciate this suggestion and believe it has strengthened the transparency and applicability of the protocol.

4. Comment: Role of "critical/important" classification: It is mentioned that experts will categorize issues as "critical," "important," or "not important." How this classification will affect subsequent processes (for example, whether only "critical" and "important" issues will be synthesized for evidence and recommendations) needs to be clarified.

Answer: We thank the reviewer for this valuable comment. We agree that the role of the “critical,” “important,” and “not important” classification should be more clearly specified. We have revised the manuscript to clarify that this classification will be used as an additional prioritization tool during question selection and recommendation development. Questions rated predominantly as “critical” or “important,” and that also meet the predefined Delphi consensus threshold, will be prioritized for evidence synthesis and formulation of recommendations. Questions predominantly rated as “not important,” even if discussed during the Delphi process, will not be prioritized for full evidence synthesis unless the steering committee identifies a strong methodological or clinical reason for their retention. This clarification has been added to the Methods section.

5. Comment: Scope definition and clinical issues: It is clear that preoperative, postoperative management, and special populations are explicitly excluded. However, the scope of "intraoperative decision-making" is very broad. It is recommended that the authors provide 1–2 proposed PICO questions in the main text or supplementary materials to help readers better understand the specific concerns of the guidelines.

Answer: We thank the reviewer for this helpful suggestion. We agree that greater transparency regarding the proposed clinical questions would help readers better understand the scope of the guideline. In response, we have added the preliminary list of candidate clinical questions formulated by the Steering Committee as supplementary material and have cited this file in the manuscript. We believe this addition clarifies the specific intraoperative issues under consideration while preserving the readability of the main text.

6. Comment: Evidence synthesis plan: The possibility of network meta-analysis (NMA) is mentioned. Given that comparative studies of laparoscopic cholecystectomy may lack directly comparable randomized controlled trials, the feasibility of conducting NMA needs to be assessed. The authors should clarify under what conditions (such as at least one common comparative group, study homogeneity, etc.) NMA will be considered; otherwise, it should be clearly stated that primarily paired meta-analysis or narrative summary will be conducted.

Answer: We thank the reviewer for this important comment. We agree that the feasibility of network meta-analysis should be more clearly specified. We have revised the manuscript to clarify that pairwise meta-analysis will be the preferred quantitative synthesis approach whenever studies are sufficiently comparable and address the same direct comparison. Network meta-analysis will only be considered if the available evidence forms a connected network with at least one common comparator and if the assumptions of clinical and methodological comparability, as well as transitivity, are judged to be reasonable. If these conditions are not met, evidence will be summarized through pairwise meta-analysis when appropriate or by narrative synthesis following SWiM guidance. This clarification has been added to the Methods section.

7. Comment: Data availability statement: The current statement "Deidentified research data will be made publicly available when the study is completed and published." is too vague. For a protocol, it should specify what types of data will be shared upon completion (for example, anonymized response data from the Delphi survey, extracted data tables from the systematic review, GRADE evidence profile tables, etc.), and the planned sharing platforms (such as OSF, Figshare, or as supplementary files to the article). This aligns with best practices in open science.

Answer: We thank the reviewer for this helpful comment. We agree that the original data availability statement was too general for a protocol. We have revised the manuscript to specify the types of materials that will be shared upon completion of the study, including anonymized Delphi response data, extracted evidence tables from the systematic reviews, and GRADE evidence profile and Summary of Findings tables, when applicable. We also clarified that these materials will be made publicly available through the Open Science Framework and/or as supplementary files accompanying the final publication, in accordance with open science principles and journal requirements.

8. Comment: Ethics statement: Currently listed as "N/A." However, Delphi research involves collecting data and opinions from expert participants, which typically requires ethical review exemption or approval. The authors should confirm and supplement the corresponding ethical statement (for example, stating that the study has received approval or exemption from the ethics committee of XX institution, or that it does not require ethical approval because it falls under methodological research for guideline development, and clarify the reasons).

Answer: We thank the reviewer for this important observation. We agree that the previous ethics statement was insufficient and required clarification. The manuscript has been revised accordingly. This protocol was assessed by the corresponding institutional ethics committee and was approved through an expedited review process (approval code: CEISH-2026051; Minutes No. 040-2026). The study constitutes a low-risk methodological guideline-development project involving voluntary participation of expert panelists, with no patient enrollment, clinical intervention, or collection of sensitive personal data. In addition, Delphi responses were collected and analyzed in deidentified form.

9. Comment: Critique of existing guidelines: Pages 13–14 mention that existing guidelines "lack methodological rigor." This assertion needs to be supported more cautiously and specifically. The cited references 20 and 21 (Agresta et al., 2015; Romero et al., 2021) are also consensus-based guidelines or Delphi studies. It could be pointed out that they have shortcomings in comprehensiveness or coverage of certain technical details, rather than broadly criticizing their methodological rigor unless there is clear assessment (such as using the AGREE II tool) to support this claim.

Answer: We thank the reviewer for this thoughtful comment. We agree that our original wording was too broad and could be interpreted as an unsupported methodological judgment regarding previous consensus-based guidelines. Our intention was not to dismiss these efforts, but rather to highlight that existing documents do not comprehensively address the full range of intraoperative technical and decision-making aspects that this procedure guide aims to cover. We have therefore revised the Discussion section to adopt a more cautious and specific wording, focusing on limitations in scope, comprehensiveness, and coverage of technical details rather than making a general statement about methodological rigor.

10. Comment: Limitations: As a protocol, the discussion section should include a discussion of the potential limitations of this protocol design. For example: reliance on published literature may not cover all unpublished surgical techniques; although the Delphi expert panel is international, it may still not represent practice differences across all regions; after the guidelines are developed, their actual adoption and implementation effects need to be validated by subsequent research.

Answer: We thank the reviewer for this valuable suggestion. We agree that the Discussion section should acknowledge the potential limitations of the protocol design itself. In response, we have revised the manuscript to include a paragraph outlining key anticipated limitations, including the reliance on published evidence, the possibility that the expert panel may not fully capture all regional practice variations despite its international composition, and the need for future research to evaluate implementation, uptake, and real-world impact after the guideline is developed.

**Reviewer 3**

1. Comment: Move all of the contributors to a small table format and attach as supplementary material.

Answer: We thank the reviewer for this helpful suggestion. We agree that presenting the contributors in a table format as supplementary material improves the readability of the main manuscript. In response, we have removed the detailed contributor listing from the main text and included it in S2 Appendix.

2. Comment: Clarity on conflict management - describe how conflicts will be addressed, monitored, and managed.

Answer: We thank the reviewer for this important suggestion. We agree that the protocol should describe more explicitly how conflicts of interest will be addressed, monitored, and managed during the guideline development process. In response, we have revised the manuscript to clarify that all members of the guideline development process will be asked to disclose potential financial and non-financial conflicts of interest before participation. These disclosures will be reviewed by the coordinators and steering committee, documented, and considered when assigning roles in question formulation, evidence synthesis, and recommendation development. When deemed relevant, participants with potential conflicts may be restricted from voting or decision-making on specific questions or recommendations. This information has now been added to the Methods section.

3. Comment: Clarify how or if AI outputs will be validated by human information specialists.

Answer: We thank the reviewer for this important comment. We agree that human oversight of any AI-assisted output should be clearly stated. The manuscript has been revised to clarify that preliminary search strings drafted with the support of generative artificial intelligence will be reviewed, refined, and validated by the investigators before implementation in the databases, and that no AI-generated output will be used directly without human verification.

4. Comment: Standardizing ALL intra-op decisions seems unmanageable but possibly define a tighter scope of boundaries (ie. use of cholangiography, bailout methods like subtotal or fenestration, or different phases of the procedure).

Answer: We thank the reviewer for this thoughtful comment. We agree that the original wording may have suggested an overly broad or unmanageable scope. Our intention is not to standardize every possible intraoperative decision, but rather to provide structured, evidence-based guidance for key intraoperative domains that are most relevant to the safe and effective performance of laparoscopic cholecystectomy. In response, we have revised the manuscript to clarify that the guideline will focus on predefined intraoperative phases and priority technical decision points, including operative exposure, dissection strategies, use of adjuncts such as intraoperative cholangiography when relevant, and bailout approaches in difficult scenarios. We believe this clarification better defines the scope and feasibility of the project.

5. Comment: 50% participation seems too low and should be more around 70% for validity

Answer: We thank the reviewer for this comment. We acknowledge that higher participation rates are desirable in Delphi processes. However, we respectfully chose a predefined threshold of ≥50% participation in order to balance methodological rigor with the practical challenges of maintaining engagement in a relatively large and geographically diverse expert panel. Our aim is to ensure broad representation while minimizing the risk of excessive attrition across rounds. Importantly, this participation threshold will not be used in isolation, as consensus will additionally require ≥80% agreement among respondents. Reminder notifications will be sent during each Delphi round to optimize participation, and descriptive statistics will be used to assess the stability of responses between rounds. We have therefore decided to retain the predefined threshold as stated in the protocol.

6. Comment: How will unresolved items be handled? what would criteria be to drop, modify or differ questions/recommendations?

Answer: We thank the reviewer for this important comment. We agree that the handling of unresolved items should be described more explicitly. We have revised the manuscript to clarify that items not reaching consensus after a Delphi round will be reviewed by the coordinators and steering committee together with the accompanying free-text comments. Questions or recommendations may be modified when comments indicate problems of wording, ambiguity, overlap, or scope. Items may be dropped if they remain below the predefined consensus threshold and are considered to have limited relevance or insufficient support from the panel. Items of persistent clinical relevance that do not achieve consensus after two rounds may be retained as optional statements or deferred for future updates of the guideline. This clarification has been added to the Methods section.

7. Comment: Are there public or private stakeholders?

Answer: No formal public or private stakeholder groups were involved in this protocol. Participation was limited to the coordinating team, steering committee, and invited experts selected according to their clinical, academic, and methodological expertise relevant to laparoscopic cholecystectomy.

8. Comment: Provide rationale on size and composition of the expert panel

Answer: We thank the reviewer for this important comment. We agree that further clarification regarding the size and composition of the expert panel strengthens the methodological transparency of the study. The target panel size of approximately 60 participants was selected to balance diversity and feasibility, ensuring broad representation across institutions, healthcare settings, and geographic regions while maintaining adequate participation throughout the Delphi rounds.

Experts were invited based on recognized clinical expertise in laparoscopic cholecystectomy and/or hepatobiliary surgery, academic and/or research experience, years of professional practice, and involvement in surgical teaching or quality improvement initiatives. This composition was considered appropriate because hepatobiliary and experienced laparoscopic surgeons are directly involved in the clinical and technical aspects addressed by the guideline. Methodological support was provided by team members with experience in evidence synthesis and guideline development, and anesthesiologists were included for questions related to perioperative analgesia and anesthetic management.

We have revised the Methods section to explicitly describe the rationale for panel size and composition.

**Reviewer 4**

1. Comment: The authors plan to develop guidelines for safe laparoscopic cholecystectomy as most of the current guidelines focus only on bile duct injury prevention. The plan of developing guidelines is well formulated and it is in accordance with the current practices for development of similar guidelines.

Answer: We sincerely thank the reviewer for this positive assessment of our protocol and for recognizing that the planned guideline development process is aligned with current methodological standards for guideline development. We appreciate the reviewer’s supportive comments.

**Reviewer 5**

Comment: Though there is vast knowledge and guidelines available on the topic of the study, the authors are right in pointing out the lack of globally applicable and holistic guidelines that address the subject of laparoscopic cholecystectomy. However, the proposed partcipants from membership of surgical society from a single country may not adequately address the topic for global consumption. At most, the study as proposed would serve as a national or regional guideline on the subject. Further, the authors mention recruiting external experts via invitation by a steering committee. The criteria for inclusion of international experts is not well elaborated. The authors also mention ensuring broad representation 'across surgical specialties' yet the topic is a guideline on a subspecialized area of general surgery. The aim should not be broad representation but subspecialized representation from the HPB/general surgeons with considerable experience on the subject. I recommend that the authors tap into the memebrship of cross-border or international bodies such as IHBA or ILTS for the study to achieve the objectives and serve as global guideline or protocol on the subject.

Answer: We thank the reviewer for this thoughtful and constructive comment. We fully agree that, to achieve global applicability, it is essential to ensure adequate international representation and appropriate subspecialty expertise.

Regarding panel composition, although a substantial proportion of participants are members of the Colombian Association of Surgery, the panel is not limited to a single country. External experts are being actively invited by the steering committee based on predefined criteria, including recognized expertise in laparoscopic cholecystectomy and/or hepatobiliary surgery, academic and research experience, years of clinical practice, and involvement in surgical education or quality improvement initiatives. The intention is to ensure that participants are directly involved in the technical and clinical aspects addressed by the guideline.

We also agree with the reviewer that the focus should be on subspecialized expertise rather than broad representation across unrelated surgical disciplines. The manuscript has been revised to clarify that the panel primarily includes hepatobiliary and experienced laparoscopic surgeons, rather than a wide range of surgical subspecialties.

In addition, we acknowledge the importance of broader international collaboration. The steering committee is actively working to include experts from different regions and institutions, and we recognize that engagement with international surgical societies represents an important opportunity to further strengthen the global applicability of the guideline. This will also be considered in future phases, including dissemination and updates.

We have revised the Methods section to better clarify the criteria for selection of international experts and the intended scope of representation.

**Reviewer 6**

1. Comment: Clarification of Added Value Compared to Existing Guidelines The manuscript states that current guidelines insufficiently address intraoperative technical conduct. While this may be true, the manuscript would benefit from a clearer and more structured comparison with major existing guidelines (e.g., WSES 2020 guidelines, multi-society safe cholecystectomy consensus, prior consensus-based recommendations). To strengthen the justification for this initiative, the authors should: • Explicitly outline how the proposed guideline differs in scope. • Clarify whether it will cover a broader intraoperative workflow than existing initiatives. • Specify how methodological rigor (e.g., systematic use of GRADE for all recommendations) distinguishes this effort. A concise comparative table may improve clarity and strengthen the manuscript’s rationale.

Answer: We thank the reviewer for this constructive suggestion. We have revised the Discussion section to more clearly distinguish the proposed guideline from existing initiatives. Specifically, we now clarify that this procedure guide is intended to cover a broader intraoperative workflow, including access and setup, exposure, dissection strategies, adjunctive intraoperative techniques, documentation of safety steps, and bailout approaches. We also emphasize the structured methodological framework of the project, including PICO-based questions, systematic evidence synthesis, Delphi consensus, and GRADE assessment for certainty of evidence and strength of recommendations.

2. Comment: Role of ChatGPT in Systematic Review Methodology

The manuscript indicates that systematic literature searches will be performed with assistance from ChatGPT 5.0. While innovative, this statement requires clarification to avoid concerns about reproducibility and methodological rigor.

The authors should clarify: • Whether ChatGPT will assist only in drafting preliminary search strategies. • Whether final search strategies will be validated by a trained information specialist or librarian. • How transparency and reproducibility will be ensured. • Whether complete search strategies will be provided as supplementary material.

Providing this clarification will strengthen confidence in the systematic review methodology.

Answer: We thank the reviewer for this important comment. We agree that the role of ChatGPT in the search process required clarification. We have revised the Methods section to specify that generative AI will only be used, when needed, to assist in drafting preliminary search terms and Boolean combinations. Final search strategies will be developed, reviewed, refined, and validated by the investigators, with methodological oversight from team members experienced in evidence synthesis and, when available, consultation with an information specialist or librarian. No AI-generated search strategy will be implemented without human verification. To ensure transparency and reproducibility, complete final search strategies for each database will be reported in supplementary material in the final guideline.

3. Comment: Delphi Process and Participation Threshold

The defined consensus threshold (≥50% participation and ≥80% agreement) should be justified with reference to Delphi methodology standards. In particular, the rationale for a 50% participation threshold warrants explanation, as participation rates may influence representativeness.

The authors should also clarify how attrition between Delphi rounds will be handled and whether subgroup analyses (e.g., geographic representation, practice setting) are planned to ensure balanced input.

Answer: We thank the reviewer for this important comment. We agree that the participation threshold and handling of attrition should be more clearly justified. We have revised the Methods section to clarify that the ≥50% participation threshold was selected to balance methodological rigor with the practical challenges of conducting a multi-round Delphi process involving a large, geographically diverse international expert panel. Consensus will not be based on participation alone, but will additionally require ≥80% agreement among respondents. Reminder notifications will be sent during each round to minimize attrition, and response rates will be reported for each Delphi round. We also clarified that attrition between rounds will be documented and assessed descriptively. Where feasible, respondent characteristics such as geographic region, practice setting, and professional background will be summarized to evaluate the representativeness of the participating panel.

4. Comment: 4. Clarification of Scope Boundaries The guideline excludes preoperative and postoperative management, yet anesthesiologists are included for perioperative considerations. It would be helpful to clarify whether anesthesia-related aspects are strictly limited to intraoperative management. Including one example PICO-formulated clinical question would enhance transparency and help readers understand the intended granularity and scope of recommendations.

Answer We thank the reviewer for this helpful comment. We agree that the scope boundaries should be clarified. We have revised the Methods section to specify that anesthesia-related input will be limited to intraoperative or procedure-adjacent issues directly affecting the conduct and safety of laparoscopic cholecystectomy, such as pneumoperitoneum pressure, positioning-related physiological considerations, and selected analgesic interventions performed during the operation. The guideline will not address broader preoperative anesthetic assessment or postoperative pain management pathways.

To improve transparency regarding the intended granularity and scope of the recommendations, we have also provided the preliminary list of candidate PICO-formulated clinical questions as supplementary material (S1 Appendix).

5. Comment: Use of Existing Systematic Reviews The manuscript proposes relying on systematic reviews published within the past five years when available. The authors should clarify: • Whether updated searches will be conducted before adopting conclusions. • How methodological quality will be assessed before incorporating existing reviews. • How overlapping or conflicting reviews will be managed. Clarifying these criteria will strengthen methodological consistency.

Answer: We thank the reviewer for this important comment. We agree that the approach to incorporating existing systematic reviews should be clarified to ensure methodological rigor and consistency. The manuscript has been revised accordingly.

When a recent systematic review (within the past five years) is identified, an updated literature search will be performed to capture newly published studies and ensure the currency of the evidence. The methodological quality of eligible systematic reviews will be assessed using the AMSTAR-2 tool prior to their inclusion. In cases where multiple systematic reviews address the same question, we will prioritize the most recent, comprehensive, and methodologically robust review. When substantial overlap or conflicting conclusions are identified, an umbrella review approach will be used, and discrepancies will be explored by examining differences in inclusion criteria, study selection, and risk-of-bias assessments. When necessary, primary studies will be re-evaluated to ensure consistency and completeness of the evidence base.

6. Comment: Data Availability Statement As this is a protocol without primary data generation, the data availability statement should be clearly aligned with PLOS ONE’s policy for protocols and explicitly state that no datasets were generated or analyzed at this stage.

Answer: We thank the reviewer for this helpful comment. We have revised the Data Availability Statement to align with PLOS ONE’s policy for protocol manuscripts by explicitly stating that no datasets were generated or analyzed for the present protocol. We have also specified our planned approach for data sharing after completion of the guideline, including deidentified Delphi response data, extracted evidence tables, and GRADE evidence profile and Summary of Findings tables, as applicable.

7. Comment: Minor Comments 1. “Paso 7” should be revised to “Stage 7.” 2. Minor typographical and spacing inconsistencies should be corrected. 3. Figures 1–3 would benefit from brief descriptive explanations in the main text. 4. The registration section should include the date of registration and confirm that it was prospectively registered.

Answer: We thank the reviewer for these helpful minor comments. We have corrected “Paso 7” to “Stage 7” and reviewed the manuscript for typographical and spacing inconsistencies. We have also added brief descriptive explanations for Figures 1–3 in the main text. Finally, we revised the registration statement to include the registration date and to clarify that the protocol was prospectively registered.

All corrections have been highlighted in yellow color.

We are attentive to any necessary corrections or additional comments.
