# Supplementary material for: Correction: Protocol for the development of a procedure guide on Laparoscopic Cholecystectomy: Beyond bile duct injury prevention
Source: PLoS One. 2026 Jun 18;21(6):e0352181. doi: 10.1371/journal.pone.0352181 (PMC13278389; doi:10.1371/journal.pone.0352181)
Supplement: S2 Appendix — (DOCX) [file pone.0352181.s001.docx]

**S2 appendix.** Full list of contributors and institutional affiliations.

**Coordinators:** Study conception and design, drafting of manuscript, critical revision of manuscript.

**Steering committee and participants:** Drafting of manuscript and critical revision of manuscript.

**Acknowledgements**

**^1^**Bogotá Task Force Collaboration Group

**Guidelines Development Group**

**Coordinators**

Camilo Ramírez-Giraldo

Department of General Surgery, Hospital Universitario Mayor - Méderi

Calle 24 #29-45 - 111411 Bogotá, Colombia

Tel: +57 3206770474

e-mail: [camilo.ramirezg@urosario.edu.co](mailto:camilo.ramirezg@urosario.edu.co)

ORCID: 0000-0002-1929-2299

Daniela Álvarez-León

Escuela de Medicina y Ciencias de la Salud, Universidad del Rosario

Carrera24 #63C-69 - 111411 Bogotá, Colombia

Tel: +57 3165471316

e-mail: [daniela.alvarezl@urosario.edu.co](mailto:daniela.alvarezl@urosario.edu.co)

Alejandro Karduss-López

Escuela de Medicina y Ciencias de la Salud, Universidad del Rosario

Carrera 24 #63C-69 - 111411 Bogotá, Colombia

Tel: +57 3165471316

e-mail: [alejandro.karduss@urosario.edu.co](mailto:alejandro.karduss@urosario.edu.co)

**Steering committee**

Andrés Isaza-Restrepo

ORCID iD: 0000-0002-1569-969X

andres.isaza@urosario.edu.co

Alberto Navarro-Alean

ORCID iD: 0000-0002-6382-6892

jorge.navarro@mederi.com.co

Susana Rojas-López

ORCID iD: 0009-0002-6599-3341

susana.rojas@mederi.com.co

Department of General Surgery, Hospital Universitario Mayor – Méderi, Bogotá, Colombia

Antonio Pesce

Department of Surgery, Azienda Unità Sanitaria Locale Ferrara, University of Ferrara, Via Valle Oppio, 2, 44023 Lagosanto, FE, Italy

ORCID: 0000-0002-7560-551X

José Alejandro Daza-Vergara

ORCID: 0000-0002-3810-3094

jose.daza@mederi.com.co

Luisa Fernanda Murcia-Soriano

ORCID: 0000-0003-2998-8561

luisa.murcia@mederi.com.co

Research Department. Hospital Universitario Mayor – Méderi, Bogotá, Colombia

**Participants**

Alejandro González-Muñoz

Hospital de Kennedy, Subred Sur Occidente E.S.E. Bogotá, Colombia

ORCID iD: 0000-0003-3890-3726

alegon_zalez@hotmail.com

Ana Carolina Buffara Blitzkow

Unit of General Surgery of Hospital de Clínicas da Universidade Federal do Paraná (UFPR) - Curitiba - Paraná - Brazil

ORCID iD: 0000-0003-1791-9892

anacarolina@mps.com.br

Arda Isik

Istanbul Medeniyet University, Istanbul

ORCID iD: 0000-0001-9493-4055

kararda@yahoo.com

Audrius Dulskas

National Cancer Institute, Vilnius, Lithuania and Vilnius University, Faculty of Medicine

ORCID iD: 0000-0003-3692-8962

audrius.dulskas@gmail.com

Camilo Andres Garcia Riaño

Hospital Internacional de Colombia - Fundación Universitaria FCV

ORCID iD: 0000-0001-5496-9413

camiloagarciar@gmail.com

Carlos Eduardo Rey Chaves

Pontificia Universidad Javeriana

ORCID iD: 0000-0001-6888-5595

carlosrey991@gmail.com

Danilo Osorio

Universidad del Cauca

ORCID iD: 0000-0002-1766-4722

danilof@unicauca.edu.co

Danny Conde-Monroy

LATAM AHPBA Hepatobiliary surgery fellowship

ORCID: 0000-0002-1365-4674

condedanny889@gmail.com

Diego Sierra Barbosa

Cirujano General – Profesor Universidad de La Sabana

ORCID iD: 0000-0002-0584-3897

diego.sierra@unisabana.edu.co

Ewen M Harrison

University of Edinburgh / Royal Infirmary of Edinburgh

ORCID iD: 0000-0002-5018-3066

Ewen.Harrison@ed.ac.uk

Fabio Vergara Suárez

Cirujano hepatopancreatobiliar. Hospital internacional de Colombia (HIC)

ORCID: iD: 0000-0002-67382379

fabiovergara14@gmail.com

Fabrizio D’Acapito

U.O. Chirurgia Generale e Terapie Oncologiche Avanzate, Ospedale Morgagni-Pierantoni, Forlì, Italy

ORCID iD: 0000-0001-6420-6209

fabrizioda@gmail.com

Felipe Casas Jaramillo

Cirujano general La cardio

ORCID: 0000-0002-0174-519X

fcasasj@lacardio.org

Fernando Gutiérrez Infante

Especialista en entrenamiento en cirugía hepatopancreatobiliar Unisanitas

ORCID iD: 0000-0003-4412-9802

fernandogutierrezinfante@gmail.com

Gökhan Demiral

Recep Tayyip Erdoğan University, Faculty of Medicine, Department of General Surgery

ORCID iD: 0000-0003-2807-5437

drgokhandemiral@yahoo.com

Gustavo Martinez Mier, FACS

Department of Organ Transplantation, General Surgery & Division of Research. Unidad Médica de Alta Especialidad, Hospital de Especialidades No. 14, Centro Médico Nacional “Adolfo Ruiz Cortines”, Instituto Mexicano del Seguro Social (IMSS). Veracruz 91897, Veracruz, Mexico

ORCID iD: 0000-0002-2883-9188

gmtzmier@hotmail.com

Ingrith Motta-Rincón

Departamento de Cirugía General - Hospital Naval de Cartagena - Armada Nacional de Colombia

ORCID iD: 0009-0008-8150-0408

imottarin@gmail.com

Ismael Domínguez Rosado

Departamento de Cirugía, Instituto Nacional de Ciencias Médicas y Nutrición Salvador Zubirán

ORCID iD: 0000-0002-5940-4208

Jorge David Peña Suárez

Clínica Reina Sofía, Clínicas Colsanitas

ORCID iD: 0000-0002-3516-2865

jorged.pena@urosario.edu.co

Jorge Muñoz Infante

Jefe de la Unidad de Educación. Centro Médico ISSEMYM

ORCID iD: 009-006-9167-8862

herniacentremexico@gmail.com

José Luis Quezada González

Filiación institucional: Hospital del Salvador / Clínica Bupa /Universidad de Chile - Chile

ORCID iD: 0000-0003-0722-099X

jlquezadag@gmail.com

Juan Carlos Luna Cydejko

Clínica Internacional

ORCID iD: 0000-0002-0826-6589

consultas@doctorjcluna.com

Juan Pablo Muñoz Alzate

Anesthesiology, Universidad de Antioquia. Medellín, Colombia.

ORCID iD: 0009-0001-8975-2529

juanpabloma021191@gmail.com

Laura Covelli

Hospital Universitario Mayor – Méderi, Bogotá, Colombia

ORCID iD: 0000-0002-3329-9614

lau.x.covelli@gmail.com

Lovenish Bains

Department of Surgery, Maulana Azad Medical College & Lok Nayak Hospital, New Delhi- 110002, India

ORCID iD: 0000-0002-8627-0452

lovenishbains@gmail.com

Luis Gabriel González Higuera

Hospital universitario Nacional, Bogotá, Colombia

0009-0006-0155-4859

lugagonzalezh@hotmail.com

Marcello Di Martino

Department of Health Sciences, University of Piemonte Orientale, 28100 Novara, Italy.

ORCID iD: 0000-0001-6510-7210

marcello.dimartino@uniupo.it

Marcelo A. F. Ribeiro Jr.

University of Maryland - R Adams Cowley shock trauma center - Baltimore, MD, USA

ORCID iD: 0000-0001-9826-4722

mfribeiro@som.umaryland.edu

Marco Antonio Vanegas Cabrera

Hospital Universitario Mayor – Méderi, Bogotá, Colombia

ORCID iD: 0000-0002-5298-3825

marco9109@gmail.com

María Paula Moreno Knudsen

Hospital Universitario Mayor - Méderi

ORCID iD: 0009-0005-8262-0928

mpmoreknudsen@gmail.com

Mariana Ramírez Ceballos

Fundación Liga Ama Salvar Vidas - Pereira, Risaralda.

ORCID iD: 0000-0003-1171-5397

marianarceballos@gmail.com

Mohammed A. Omar

Qena University Hospitals, Qena University, Egypt

ORCID iD: 0000-0002-2736-8097

elqefty@yahoo.com

Moisés Barrientos Rivera

Hospitales La Paz (SERMESA), Guatemala, Guatemala

ORCID: 0009-0002-7302-4663

mbarrientos63@gmail.com

Mónica Gómez González

Department of General Surgery. Hospital Universitario Mayor – Méderi, Bogotá, Colombia

ORCID: 0000-0001-9788-4970

monicac.gomez@urosario.edu.co

Mónica Parrado Delgado

Cirujana General – Profesor Universidad de La Sabana

ORCID: 0009-0005-6666-1543

monipd2490@gmail.com

Natalia Andrea Rivera Rincón

Burjeel Hospital, Abu Dhabi – General Surgery Department

ORCID: 0000-0001-9801-6809

nataliaa.rivera@outlook.com

Néstor Vega Yuil

Hospital Santo Tomas/ Facultad de Médicina-Universidad de Panamá.

ORCID iD: 0009-0001-1555-1994

nestorvy@gmail.com

Oscar Rincón Barbosa

Hospital Militar

ORCID iD: 0000-0001-9605-4254

kelmvelx@gmail.com

Rafael Arraut-Gámez

Coordinador departamento de cirugía general - Hospital universitario Evaristo García HUV, sede Cartago - Valle del Cauca

ORCID iD: 0000-0002-0264-9853

reag14@hotmail.com

Raquel Tabares-Meza

Clínica Universitaria Colombia

ORCID iD: 0000-0002-3153-5695

raquel.tabares@gmail.com

Roberto Cirocchi

University of Perugia

ORCID iD: 0000-0002-2457-0636

roberto.cirocchi@unipg.it

Rocío Anula Fernández

Servicio de Cirugía. Hospital Clínico San Carlos, Instituto de Investigación Sanitaria San Carlos. Madrid, España

ORCID iD: 0000-0001-8112-1530

ranula@ucm.es

Saul Vargas-Rubiano

Hospital Universitario mayor – Méderi

saulvargas17@gmail.com

Sebastián Benavides Largo

Pontificia Universidad Javeriana, HUSI

ORCID iD: 0000-0002-3940-0162

sbenavides@husi.org.co

Sebastian Sierra Sierra

Universidad CES - Clinica CES, Medellín, Colombia

ORCID iD: 0000-0002-3253-028X

sebastiancirugia@gmail.com

Sergio Sanz

Universidad del Tolima - Hospital Federico Lleras

ORCID iD: 009-000-4996-6007

sersanz@yahoo.es

Shiva Jayaraman

St. Joseph's Health Centre - Unity Health Toronto; University of Toronto

ORCID iD: 0009-0004-4487-3521

Shiva.Jayaraman@unityhealth.to

Vicente E. Rodríguez-Maya

Hospital Clínica San Agustín, Loja - Ecuador

ORCID iD: 0000-0002-2692-0724

vicenterodma@gmail.com

Victor Manuel Quintero Riaza

Jefe seccion cirugia general, Hospital universitario Pablo Tobon Uribe Medellin

ORCID iD: 0000-0002-3889-6397

vquintero@hptu.org.co

Vishal Gupta

Department of Surgical Gastroenterology, All India Institute of Medical Sciences (AIIMS), Bhopal, MP 462020, INDIA

ORCID iD: 0000-0003-3574-1805

drvggis@gmail.com

Vishal G Shelat

Senior Consultant, Department of General Surgery, Tan Tock Seng Hospital, Singapore, 308433

ORCID iD: 0003-3988-8142

vgshelat@gmail.com
